# Are changes in physical activity associated with depression? A follow-up study of 1,950 individuals

**DOI:** 10.31744/einstein_journal/2025AO1128

**Published:** 2025-03-14

**Authors:** Luana de Lima Queiroga, Oskar Grau Kaufmann, Raphael Mendes Ritti-Dias, Carlos André Minanni, Rafael Mathias Pitta, Nelson Wolosker

**Affiliations:** 1 Programa de Pós-Graduação em Ciências da Saúde Faculdade Israelita de Ciências de Saúde Albert Einstein Hospital Israelita Albert Einstein São Paulo SP Brazil Programa de Pós-Graduação em Ciências da Saúde, Faculdade Israelita de Ciências de Saúde Albert Einstein, Hospital Israelita Albert Einstein, São Paulo, SP, Brazil.; 2 Programa de Pós-Graduação em Ciências da Reabilitação Universidade Nove Julho São Paulo SP Brazil Programa de Pós-Graduação em Ciências da Reabilitação, Universidade Nove Julho, São Paulo, SP, Brazil.; 3 Faculdade de Medicina Universidade de São Paulo São Paulo SP Brazil Faculdade de Medicina, Universidade de São Paulo, São Paulo, SP, Brazil.; 4 Hospital Israelita Albert Einstein São Paulo SP Brazil Hospital Israelita Albert Einstein, São Paulo, SP, Brazil.

**Keywords:** Exercise, Exercise therapy, Depression, Lifestyle, Sedentary behavior, Surveys and questionnaires

## Abstract

**Objective:**

To evaluate the association between changes in physical activity and depression in 1,950 Brazilians.

**Methods:**

This follow-up study included 1,950 Brazilians, aged ≥18 years, of both sexes, who participated in a health screening initiative between 2008 and 2022. Physical activity levels were evaluated using the International Physical Activity Questionnaire at baseline and follow-up, and groups related to changes in physical activity were created. Depression was assessed using the Beck Depression Inventory. Clinical data and behavioral profiles were also analyzed. Comparative and logistic regression models were used.

**Results:**

The multivariate analysis demonstrated that persistently active individuals had reduced depression (odds ratio [OR], 0.70, 95%CI= 0.55-0.91, p=0.006). Individuals who became active and those who became less active were not associated with a reduction in depression (respectively, OR= 0.81, 95%CI= 0.63-1.05, p=0.109 and OR= 1.11, 95%CI= 0.82-1.51, p=0.481).

**Conclusion:**

Persistent activity was an independent, strong protective factor against depression in 1,950 Brazilians. Furthermore, becoming active or less active was not associated with depression in the same population.

## In Brief

This study evaluated the association between changes in physical activity and depression among 1,950 Brazilians. Individuals who increased their physical activity and those who decreased it were not associated with reduced depression. Conversely, being consistently active was identified as an independent and significant protective factor against depression in this population.

## Highlights

■ Persistently active individuals have strong independent protective factors against depression.

■ BMI (kg/m) is an independent risk factor for depression.

■ Individuals with depression at follow-up were older than those without depression.

■ Perceived stress is a strong independent risk factor for depression.

## Graphical abstract

**Figure:**
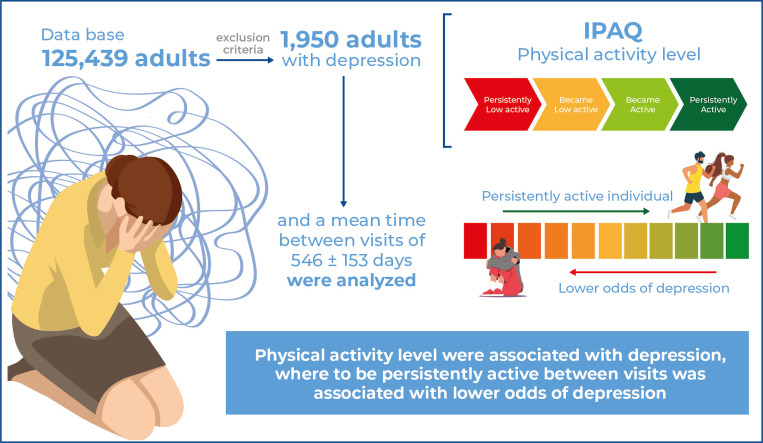

## INTRODUCTION

Depression is recognized as the leading cause of mental health-related illnesses and disabilities worldwide, affecting approximately 280 million people.^1^ It is also associated with decreased quality of life, systemic inflammation^2 , 3^and premature mortality due to other illnesses^4^ in addition to suicide.^5^

Depression is treated in primary care settings using psychological and pharmacotherapeutic interventions.^1^ In addition, lifestyle interventions, specifically physical exercise, have been used as complementary treatments for depression across various genders, age groups, and chronic health conditions.^6 - 8^ In this sense, physical activity (PA) has been associated with a reduced risk of depression.^1 - 9^ España-Romero et al.^10^ conducted a longitudinal analysis over 6.1 years to examine the relationship between PA and depression among 5,110 male and female participants aged 20 to 83 years from the Aerobics Center Longitudinal Study. Physical activity levels were determined using surrogate activity and physical fitness measures based on hemodynamic and behavioral data, while depression was evaluated using the Epidemiological Studies Depression Scale. Active individuals (more than 150 minutes per week in follow-up) reduced their risk of depression by 22% compared to sedentary individuals (less than 10 minutes per week). However, it was not possible to identify the same association in less active individuals during follow-up (OR= 1.12, 95%CI = 085-1.47, p>0.001). Similarly, Mikkelsen et al.^11^ conducted a longitudinal study over 26 years, observing an association between PA and depression in 10,625 Danish adults and older adults from the Copenhagen City Heart Study. Physical activity was assessed using a single question, and the classification of PA levels varied according to the analysis periods (1976-1978, 1981-1983, and 1991-1994). After adjusting for confounding variables, including income, education, smoking, alcohol consumption, body mass index (BMI), and the presence of diseases, it was found that lower PA levels during the analysis periods were associated with a higher risk of depression in women (OR= 1.80, 95%CI= 1.29-2.51). In men, this association was not statistically significant.

Although previous longitudinal studies have demonstrated an association between PA and depression, the relationship between PA levels and depression remains unclear. Additionally, the longitudinal effects of changes in PA levels among adults and older individuals across both sexes with depression have been assessed in the literature using standardized questionnaires that evaluate the volume and intensity of physical activity in leisure, commuting, occupation, and daily life. These questionnaires classify individuals into different levels of physical activity, complicating the understanding of the multifactorial nature of these variables in relation to depression over time.

## OBJECTIVE

To evaluate the association between changes in physical activity and depression using standardized questionnaires in a sample of 1,950 Brazilian males and females.

## METHODS

The primary aim of this study was to identify the association between changes in PA and depression in Brazilian adults and older individuals of both sexes. Health data were collected from a large cohort of men and women aged 18 years or older who participated in health screening initiatives at the Center for Preventive Medicine at *Hospital Israelita Albert Einstein* between 2008 and 2022. A waiver of informed consent was obtained.

Initially, data from 125,439 adults of both sexes were included in the database. Only the most recent health screening was considered for individuals with duplicate data, *i.e.* , more than two health screenings. Participants who underwent only one health screening were excluded. Similarly, we considered an evaluation period of 300 to 800^12^ days between the first and second health screenings and excluded data outside this range. If patients underwent three or more health screenings during the study period, only the first and most recent screenings were evaluated. We also excluded individuals with missing PA and depression data, as well as those without any degree of depression at baseline (Beck Depression Inventory score <14). Finally, data on PA and depression from 1,950 adults over 18 years of age, with a mean time between visits of 546±153 days, were analyzed.

The Ethics Committee of *Hospital Israelita Albert Einstein* approved this study (CAAE: 69234123.6.0000.0071; # 6.204.766).

### Clinical data

Weight was measured using the InBody 230 equipment (Ottoboni^®^), and height was measured using a stadiometer. Subsequently, the BMI was calculated using the formula weight/height^2^.^12^

Blood pressure was assessed in accordance with the recommendations of the American Heart Association.^13^

Information regarding the presence of systemic arterial hypertension, diabetes, dyslipidemia, tobacco use, and medication use was obtained from the medical records.

Biomarkers, including glycated hemoglobin (HbA1C%), LDL, HDL, total cholesterol (mg/dL), triglycerides (mg/dL), uric acid (mg/dL), and ultrasensitive C-reactive protein (mg/dL) were collected after overnight fasting, following standardized criteria to meet the quality standards established by the Brazilian Ministry of Health.^12^

### Behavioral data

Lifestyle factors were assessed using standardized questionnaires. Alcohol consumption was assessed using the Alcohol Use Disorders Identification Test (AUDIT),^14^ and perceived stress was measured using the Perceived Stress Scale (PSS-10).^15^

### Physical activity and sedentary behavior

Physical activity levels and sedentary behavior (total sitting time and screen time during the week and weekend) were assessed using the International Physical Activity Questionnaire (IPAQ).^16^ The IPAQ provides information on volume (time spent), intensity (light, moderate, and vigorous), and sedentary activity during a typical week and classifies subjects into four levels: highly active, active, low active, and sedentary. Participants who engaged in at least 30 minutes of vigorous physical activity for at least 5 days per week, those who engaged in at least 20 minutes of vigorous physical activity for at least 3 days per week, or those who engaged in moderate physical activity and/or walking for at least 30 minutes on at least 5 days per week were classified as highly active. Individuals who engaged in at least 20 minutes of vigorous physical activity for at least 3 days per week or any type of physical activity for at least 150 minutes per week spread out over at least 5 days were considered active. Individuals who reported engaging in physical activity but did not meet the above criteria were classified as having low physical activity. Participants who reported no physical activity were classified as inactive.^16^ Based on these results, the highly active and active groups were considered active, while the low-activity and sedentary groups were considered low-activity. Finally, four groups were created for the longitudinal assessment of changes in physical activity levels as follows: Group 1) persistently low activity-individuals who were classified as low activity at baseline and follow-up; Group 2) became low activity-individuals who transitioned to low activity at follow-up; Group 3) became active-individuals who transitioned to active at follow-up; and Group 4) persistently active-individuals who were active at both baseline and follow-up.

### Depression

The presence and severity of depression were assessed using the Brazilian-Portuguese version of the Beck Depression Inventory-II.^17^ Psychologists administered the questionnaire, which consisted of items referring to the previous 15 days. The questions were rated on an ordinal scale ranging from 0 to 3, with a total score ranging from 0 to 63. In this study, the presence of depression was defined as a score of ≥14 points, including mild, moderate, and severe depression, while the absence of depression was defined as a score of <14 points. At baseline, all participants in this study exhibited depression (≥14 points).

When previous diagnoses of depressive disorders were self-reported by patients, the team of psychologists contacted the relevant professionals. All patients were questioned regarding the use of antidepressants and anxiolytic medications and were evaluated at baseline and follow-up for all variables. Doctors and a multidisciplinary team-including nutritionists, physical education professionals, psychologists, physiotherapists, and nurses-advised patients experiencing clinical and behavioral changes to modify their lifestyle habits to enhance their health conditions. Patients with depression were instructed to initiate or continue professional follow-up.

We compared clinical and behavioral data regarding follow-up in patients with depression. A comprehensive logistic regression analysis was conducted to examine all factors associated with depression ( Table 1S, Supplementary Material ). Finally, we performed a stepwise backward multiple logistic regression analysis, focusing exclusively on the variables associated with statistically significant risk or protective factors for depression.

### Data analysis

The Shapiro-Wilk test was used to assess the normality of the data. Categorical variables were presented as frequencies and percentages, while numerical variables were presented as means and standard deviations. The χ^2^ test was used to compare categorical variables, denoted as the χ^2^ test. To compare numerical variables, we used Student’s *t* -test. A logistic regression model was applied based on the presence or absence of depression. Statistical significance was set at p<0.05. Adjusted odds ratios and 95% confidence intervals (95%CIs) were obtained using the logistic regression model. All statistical analyses were performed using SPSS for Windows (version 24.0; IBM Corp., Armonk, NY, USA).

## RESULTS

During the follow-up period, 756 (38.8%) participants were diagnosed with depression. Table 1 presents a comparison of clinical, demographic, and behavioral data related to changes in depression. Individuals diagnosed with depression at follow-up were older (42.10±8.76 *versus* 41.22±8.97 years, p=0.033) and had higher levels of ultrasensitive C-reactive protein (0.3±0.5 *versus* 0.2±0.4mg/dL, p=0.007) and uric acid (0.95±0.40 *versus* 0.94±0.16mg/dL, p=0.002) compared to those without depression at follow-up. In contrast, individuals without depression at follow-up exhibited higher HbA1C levels (5.5±0.7% *versus* 5.4±0.5%, p<0.004) than individuals with depression.

**Table 1:** Comparison of clinical, demographic and behavior data in relation to changes in depression at follow-up

Variables Mean±SD (n)	Depression			
	Absent	Present	Total	p value
Age (years)	41.22±8.97 (756)	42.10±8.76 (1,194)	41.76±8.85 (1,950)	0.033*
BMI (kg/m^2^)	26.45±4.47 (1,193)	26.81±4.49 (757)	26.59±4.48 (1,950)	0.082
Week sitting time (hours)	8.8±2.7 (96)	9.0±3.0 (84)	8.9±2.8 (180)	0.605
Weekend sitting time (hour)	5.8±2.8 (95)	5.4±2.8 (83)	5.6±2.8 (178)	0.421
TC (mg/dL)	190.9±35.9 (1,191)	192.3±36.7 (755)	191.4±36.2 (1,946)	0.392
HDL (mg/dL)	50.7±14.6 (1,190)	51.3±15.3 (755)	50.9±14.9 (1,945)	0.423
LDL (mg/dL)	115.5±32.9 (1,191)	115.9±32.9 (755)	115.7±32.9 (1,946)	0.776
TG (mg/dL)	126.2±88.0 (1,191)	128.9±84.0 (755)	127.2±86.5 (1,946)	0.503
HbAIc (%)	5.5±0.7 (909)	5.4±0.5 (601)	5.4±0.6 (1,510)	0.004*
Ultrasensitive c-reactive protein high (mg/dL)	0.2±0.4 (682)	0.3±0.5 (411)	0.3±0.5 (1,093)	0.007*
UA (mg/dL)	0.94±0.16 (718)	0.95±0.40 (427)	0.94±0.21 (1,145)	0.002*
Sex, n (%)				<0.001^#^
Male	714 (59.8)	394 (52.1)	1,108 (56.8)	
Female	480 (40.2)	362 (47.9)	842 (43.2)	
Hypertension	156 (13.1)	106 (14)	262 (13.4)	0.546
*Diabetes mellitus*	59 (4.9)	38 (5)	97 (5)	0.933
Dyslipidemia	467 (39.1)	280 (37)	747 (38.3)	0.358
Physical activity levels, n (%)				<0.001^#^
Persistently low active	442 (37.9)	329 (44.2)	771 (40.4)	
Became low active	137 (11.8)	111 (14.9)	248 (13)	
Became active	379 (23.3)	157 (21.1)	429 (22.5)	
Persistently active	314 (27)	147 (19.8)	461 (24.1)	
Tobacco use	102 (8.6)	70 (9.3)	172 (8.9)	0.614
Alcohol consumption, n (%)				0.998
Hazardous	145 (12.2)	91 (12.1)	236 (12.2)	
Moderate-severe	38 (3.2)	24 (3.2)	62 (3.2)	
Perceived stress	876 (77.4)	603 (83.4)	1,479 (79.7)	0.002^#^

* *t* -student test.; ^#^ χ^2^ test.

SD: standard deviation; BMI: body mass index; TC: total cholesterol; HDL: high-density lipid; TG: triglyceride; LDL: low-density lipids; HbAIc: glycosylated hemoglobin; UA: uric acid.

In the stepwise backward multiple logistic regression analysis, which focused only on variables associated with statistically significant risk or protection from depression, we found that BMI (kg/m^2^), female sex, and perceived stress were strong independent risk factors for depression. Conversely, age (per year) and a persistently active lifestyle were identified as strong independent protective factors against depression, as shown in table 2 .

**Table 2:** Predictors of depression

Variables	OR	95%CI	p value
Age	0.99	0.98-0.99	0.026*
BMI (kg/m^2^)	1.04	1.01-1.06	0.002*
Sex (Female)	1.45	1.18-1.77	<0.001*
Perceived stress	1.44	1.12-1.84	0.004*
Physical activity levels Persistently low active (reference)			
Became low active	1.11	0.82-1.51	0.481
Became active	0.81	0.63-1.05	0.109
Persistently active	0.70	0.55-0.91	0.006*

* Logistic regression using the backward stepwise selection method, p<0.05.

OR: odds ratio; 95%CI: 95% confidence interval; BMI: body mass index.

## DISCUSSION

This study represents the first follow-up analysis investigating the association between changes in PA levels and depression among Brazilians of both sexes aged over 18 years. Changes in PA levels were found to be associated with a decreased risk of depression. Specifically, only individuals who remained consistently active demonstrated a 30% reduction in the risk of depression (OR= 0.70, 95%CI= 0.55-0.91, p=0.006). This association was not observed in other groups.

Physical activity is related to changes in brain structure.^18^ Furthermore, PA activates the sympathetic nervous system, leading to increased levels of epinephrine and norepinephrine, as well as hormones regulated by the hypothalamic-pituitary axis, which are linked to depression.^19^ Finally, PA promotes adaptive behavioral changes and positively influences the maintenance of hippocampal volume and white matter integrity. It also activates the function of the prefrontal cortex, thus improving the efficiency of cerebral processing, delaying cognitive function degradation, and optimizing the ability to manage depressive emotions.^18 , 20^ Our findings suggest that maintaining high PA levels decreases the risk of depression over time and should be strongly encouraged in the treatment and prevention of this condition.

We observed that advanced age decreased the risk of depression (OR= 0.99, 95%CI= 0.98-0.99, p=0.026). Previous studies have demonstrated that the prevalence of depression is higher in middle-aged individuals than in older individuals of both sexes. Older adults are less likely than younger adults to endorse cognitive-affective symptoms of depression, such as dysphoria, feelings of worthlessness or guilt, sleep disorders, fatigue, psychomotor retardation, loss of interest in life, and hopelessness about the future.^21^ We hypothesize that older adults may be better equipped to manage adversities encountered in life because of their greater life experiences regarding problem situations and other aspects of life.

On the other hand, we observed that women have a 45% increased risk of depression (OR= 1.45, 95%CI= 1.18-1.77, p<0.001), according to the results of previous studies.^22 - 24^ Women appear to experience specific forms of depression-related illnesses, such as premenstrual dysphoric disorder, postpartum depression, postmenopausal depression, and anxiety, which are conditions associated with hormonal changes that contribute to the development of depression.^24^

In a similar vein, an increase of 1 kg/m^2^ in BMI was associated with a 4% rise in the risk of depression among our patients (OR= 1.04, 95%CI= 1.01-1.06, p=0.002). This finding corroborates previously presented data.^25 , 26^ Obesity elevates inflammatory markers such as CRP, interleukin-6, and TNF-α while decreasing brain-derived neurotrophic factor (BDNF), all of which are linked to the severity of depression and the development of chronic diseases. Specific symptoms associated with obesity include increased and altered appetite, insomnia, pain, decreased self-esteem, distorted self-image, reduced social interaction, and an increased risk of depression.^27^

Finally, our findings demonstrated that the presence of perceived stress increased the risk of depression by 44% (OR= 1.44, 95%CI= 1.12-1.84, p=0.004). Stressful life events can induce psychological and physiological changes, including the activation of the hypothalamic-pituitary-adrenal (HPA) axis and the sympathetic nervous system. Hyperactivity of the HPA axis is one of the most common neurobiological changes observed in patients with depression. Impulses originating from the higher cortical areas of the brain are transmitted to the hypothalamus through the limbic system^28^ and induce the release of neurotransmitters such as serotonin, norepinephrine, and acetylcholine, activating specific cells of the paraventricular nucleus in the hypothalamus and leading to the synthesis and secretion of corticotropin-releasing factors. These factors exert their effects on multiple aspects of brain function, including neuronal survival, neurogenesis, hippocampal volume, emotional events, negative feedback, and peripheral functions such as metabolism and immunity. Chronic exposure to stress induces a reduction in hippocampal volume, decreases the expression of neurotrophic factors, and inhibits neurogenesis in the adult brain.^29^

Our study was the first to investigate the association between changes in physical activity and depression in a large Brazilian cohort. We believe that cultural and environmental factors may influence these findings, including the increasing alienation and stress prevalent in industrial societies, cultural beliefs regarding human behavior, and the maintenance of high levels of physical activity. These factors are important in preventing depression, alongside other modifiable variables, such as BMI and perceived stress.

### Strengths and limitations

The strengths of the present study include: a representative cohort of individuals aged 18 years and older, with detailed demographic, health and lifestyle data collected over a period of 300 to 800 days; being the first study to investigate the association between self-initiated physical activity and depression; a more precise examination of the variables of interest, particularly PA, because of the adjustment for several confounding factors in our sample; assessments conducted by trained doctors and health professionals using questionnaires validated in the literature for the variables of interest and confounding factors; and the use of a logistic regression model, alongside clinical, demographic, and behavioral factors, to achieve a multifactorial understanding of depression in our population.

This study has several limitations that warrant consideration when interpreting the results. Physical activity was self-reported, which carries the risk of overreporting or underreporting and may introduce bias. Additionally, family history was not controlled for in relation to depression. This analysis did not account for chronic pain or sleep-related information, both of which may influence this association. Despite the temporal sequence of the study design and the inclusion of important confounding factors, it was not possible to establish a causal relationship based on our findings. Furthermore, data on race and ethnicity were not collected, as this study focused on a specific Brazilian socioeconomic sample with access to health insurance, limiting the generalizability of these findings to the entire Brazilian population. Future studies should investigate the longitudinal effects of self-initiated PA using objective measures of depression across diverse populations.

We anticipate that the results of this study will be applicable in both clinical and public health contexts. Despite its cross-sectional nature, this study can contribute to the development of preventive strategies aimed at reducing the risk of depression and improving lifestyle.

## CONCLUSION

There is an association between changes in physical activity levels and depression in Brazilian individuals of both sexes aged over 18 years. Persistently active individuals exhibited a 30% reduced risk of depression, while persistently sedentary individuals demonstrated higher depression levels at follow-up.

Risk factors associated with depression included an increased body mass index, female sex, and perceived stress. Conversely, advanced age and continuous physical activity over time were identified as protective factors against depression.

## Supplemental Material

Are changes in physical activity associated with depression?

A follow-up study of 1,950 individuals


