# Supplementary material for: Are changes in physical activity associated with depression? A follow-up study of 1,950 individuals
Source: Einstein (Sao Paulo). 2025 Mar 14;23:eAO1128. doi: 10.31744/einstein_journal/2025AO1128 (PMC11991741; doi:10.31744/einstein_journal/2025AO1128)
Supplement: Supplementary file 1 [file 2317-6385-eins-23-eAO1128-suppl01.pdf]

SUPPLEMENTARY MATERIAL

Are changes in physical activity associated with depression? A follow-up study of 1,950 individuals

Luana de Lima Queiroga, Oskar Grau Kaufmann, Raphael Mendes Ritti-Dias, Carlos André Minanni, Rafael Mathias Pitta, Nelson Wolosker

DOI: 10.31744/einstein_journal/2025AO1128

Table 1S. Predictors of depression in individuals aged ≥18 years

Variables	OR	95%CI	p value
Age	0.99	0.98-1.00	0.038*
BMI (kg/m^2)	1.04	1.01-1.06	0.003*
Sex (Male)	0.69	0.56-0.85	0.001*
Tobacco use	1.07	0.76-1.51	0.696
Perceived stress	1.43	1.12-1.84	0.005*
Alcohol consumption			
Hazardous	1.06	0.78-1.44	0.722
Moderate-severe	1.03	0.59-1.81	0.906
Physical activity levels (SS)			
AS	1.11	0.82-1.51	0.485
SA	0.82	0.63-1.05	0.112
AA	0.70	0.55-0.91	0.006*
Comorbidities	0.99	0.81-1.22	0.93

*Full multiple logistic regression, p<0.05.
OR: odds ratio; 95%CI: 95% confidence interval; BMI: body mass index; SS: dendritic at baseline and follow-up; AS: active at baseline and sedentary at follow-up; SA: sedentary at baseline and active at follow-up; AA: active at baseline and follow-up.
